# Supplementary material for: Physiological and Transcriptomic Analysis Provide Insight into Low Temperature Enhancing Hypericin Biosynthesis in Hypericum perforatum
Source: Molecules. 2021 Apr 15;26(8):2294. doi: 10.3390/molecules26082294 (PMC8071384; doi:10.3390/molecules26082294)
Supplement: Supplementary file 1 [file molecules-26-02294-s001.zip › molecules-1144993-supplementary.pdf]

Supplementary Materials

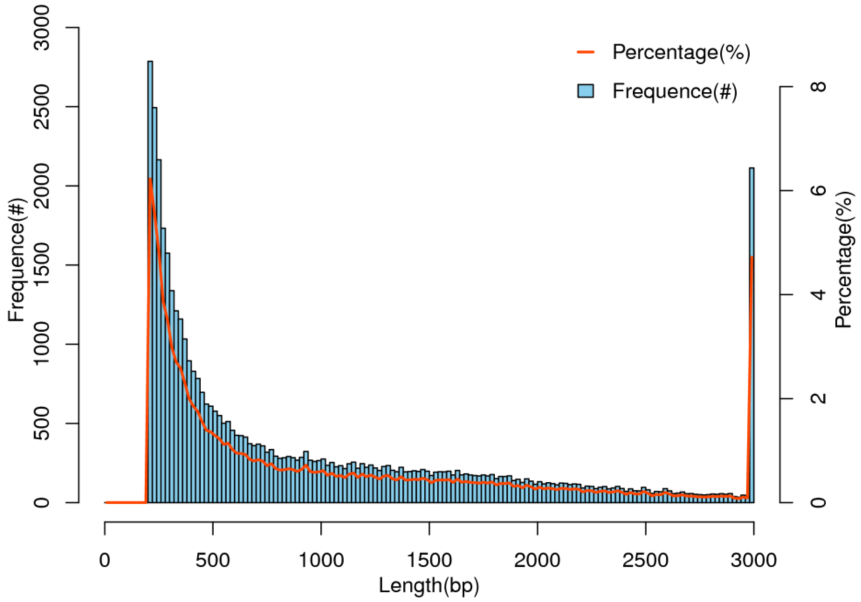

Figure S1: Length distribution of unigene.

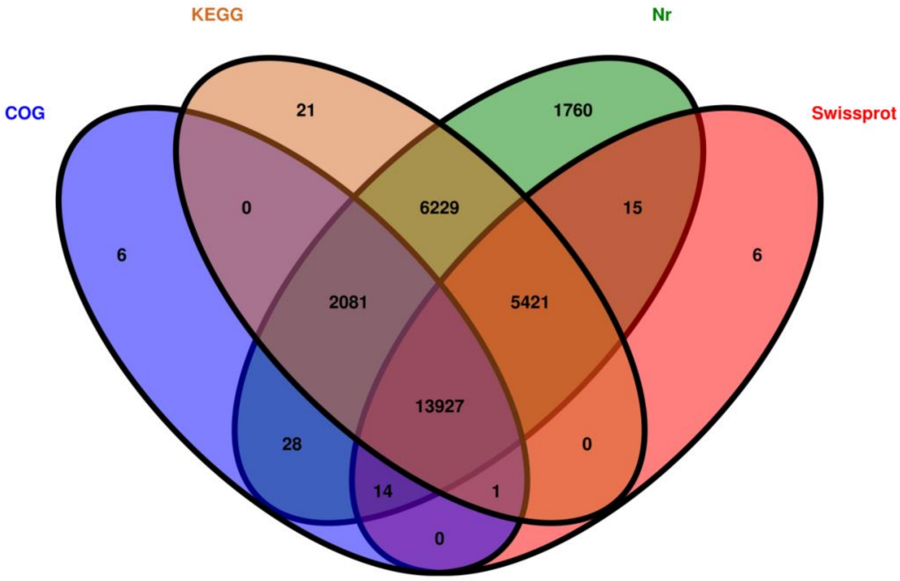

Figure S2: Basic annotation for all unigenes.

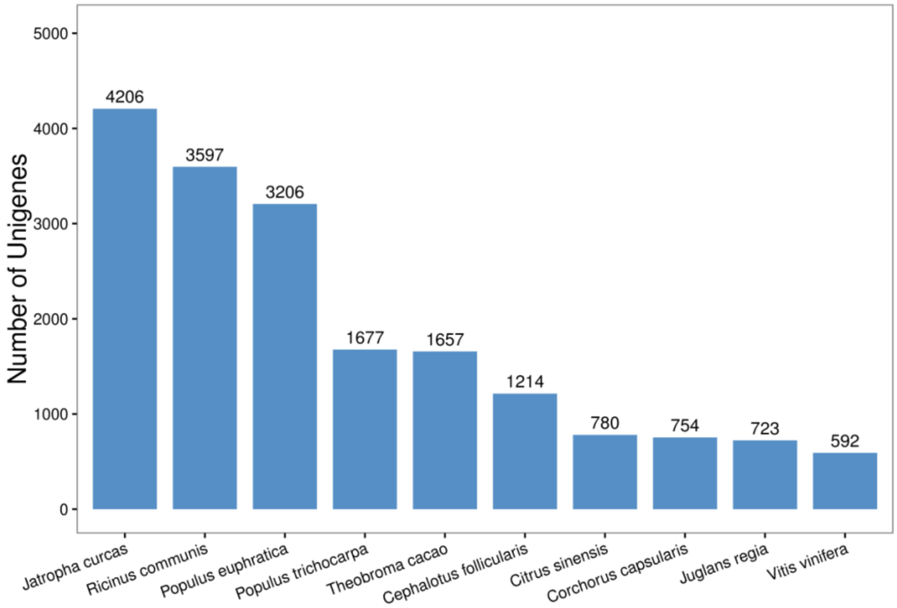

Figure S3: Top 10 plant species distribution of the total homologous sequences.

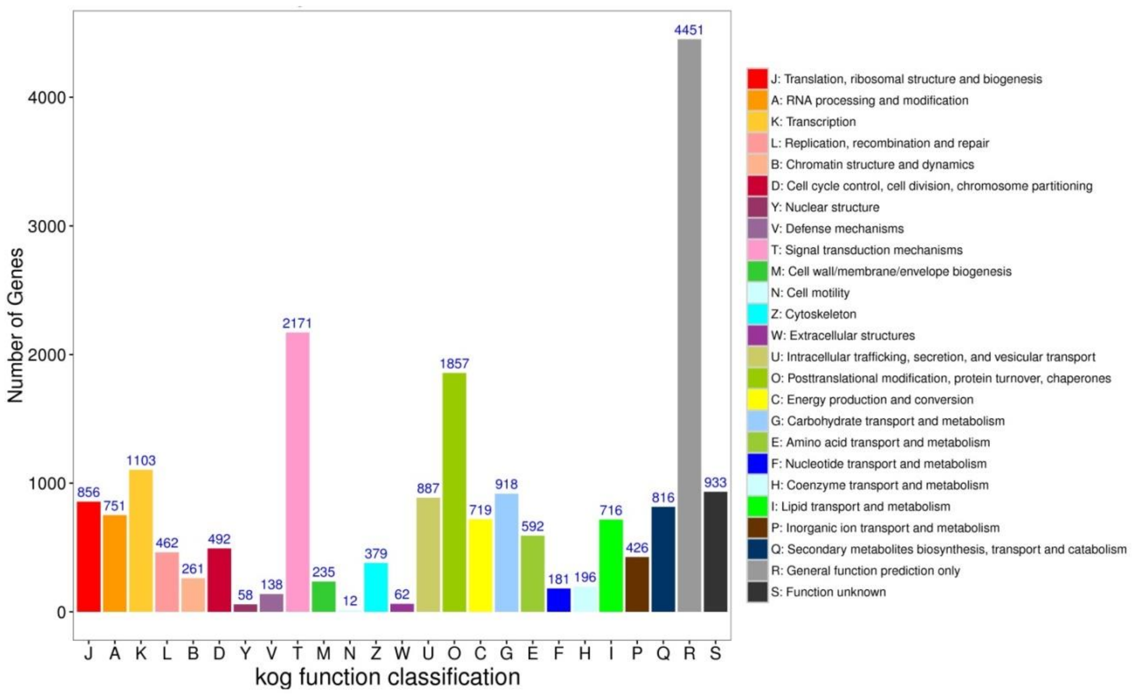

Figure S4: Distribution of unigenes in the transcriptome with KOG functional classification.

Sequences have a KOG classification among 25 categories. Note: [A] RNA processing and modification; [B] Chromatin structure and dynamics; [C] Energy production and conversion; [D] Cell cycle control, cell division, chromosome partitioning; [E] Amino acid transport and metabolism; [F] Nucleotide transport and metabolism; [G] Carbohydrate transport and metabolism; [H] Coenzyme transport and metabolism; [I] Lipid transport and metabolism; [J] Translation, ribosomal structure and biogenesis; [K] Transcription; [L] Replication, recombination and repair; [M] Cell wall/membrane/envelope biogenesis; [N] Cell motility; [O] Posttranslational modification, protein turnover, chaperones; [P] Inorganic ion transport and metabolism; [Q] Secondary metabolites biosynthesis, transport and catabolism; [R] General function

prediction only; [S] Function unknown; [T] Signal transduction mechanisms; [U] Intracellular trafficking, secretion, and vesicular transport; [V] Defense mechanisms; [W] Extracellular structures; [Y] Nuclear structure; [Z] Cytoskeleton.

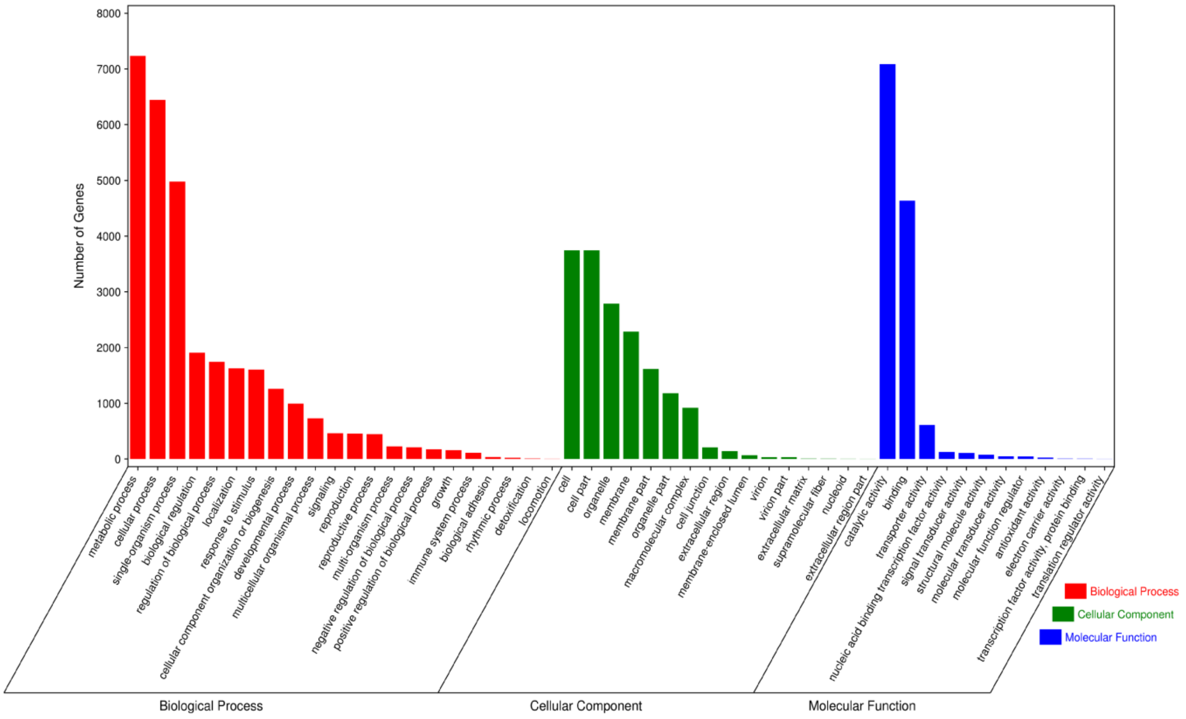

Figure S5: Functional classifications of GO terms of all assembled unigenes. Note: The results are summarized in three main categories: biological process, cellular component, and molecular function. The right y-axis indicates the number of genes in a category. The left y-axis indicates percentage of a specific category of genes.

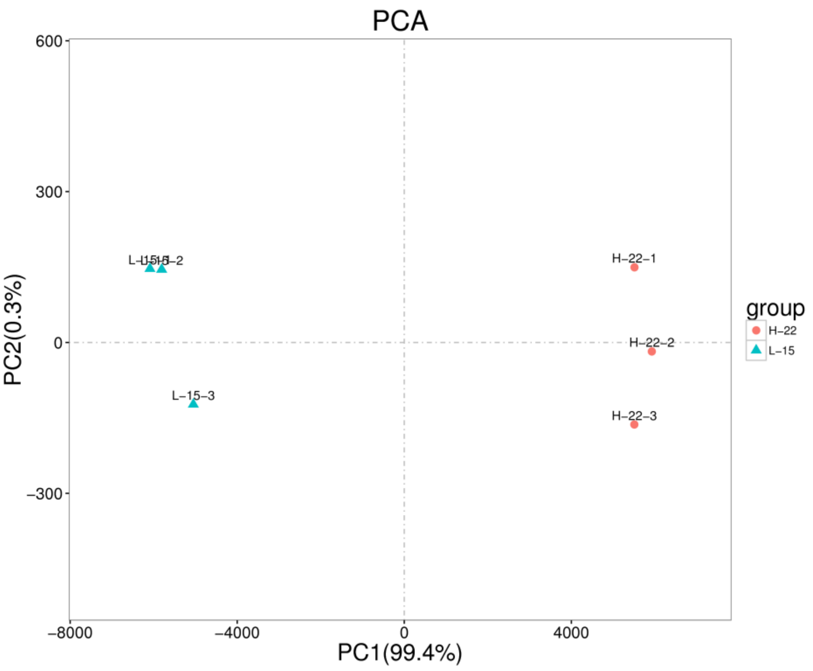

Figure S6: The PCA analysis between 15 and 22°C samples.

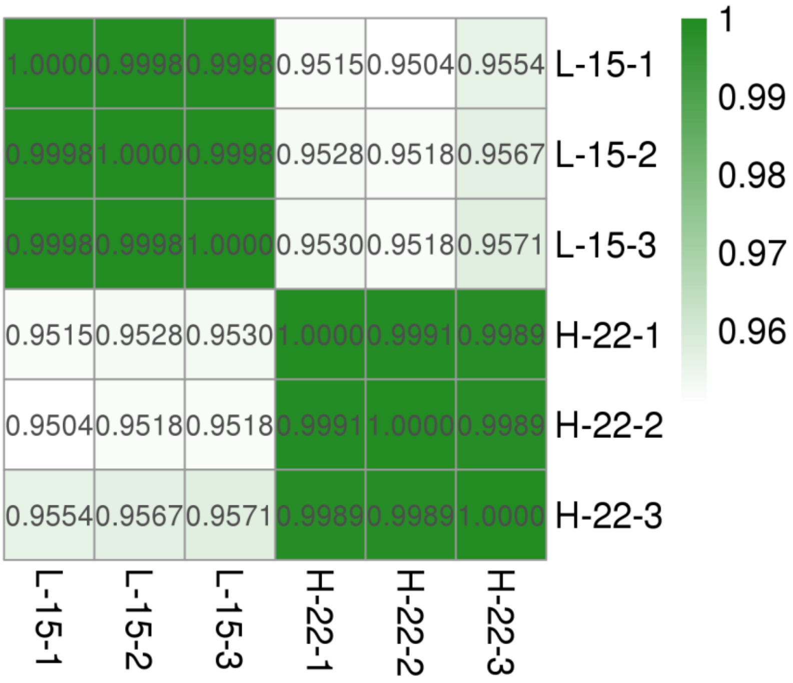

Figure S7: The correlation analysis of heat map between 15 and 22°C samples.

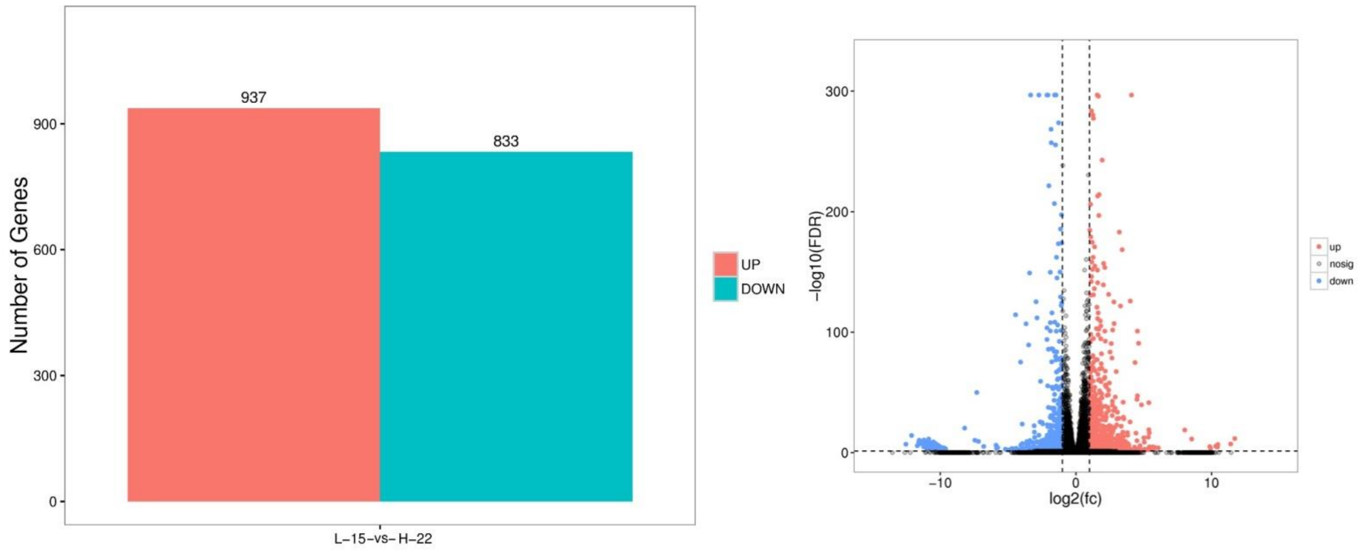

Figure S8: Number of differentially expressed genes and volcano plot when plants grown at 15 °C versus 22°C. Note: A total of 1,584 DEGs were obtained after excluding 186 repeated genes from the originally generated 1,770 genes.

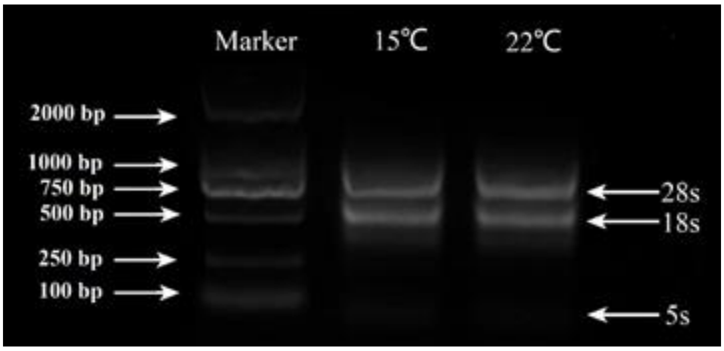

Figure S9: The quality of total RNA at 15 and 22°C was evaluated by 1.0% agarose gel electrophoresis.

Table S1: Sequences of primer employed in qRT-PCR analysis

Gene name	Primer sequences (5' to 3')	Amplicon size (bp)
ACT	Forward: ATCCTCCGTCTTGACCTTGC<br>Reverse: ACGATTTCCCGTTCTGCTGT	104
**Hyp biosynthesis (19)**		
Photosynthesis		
CAB	Forward: TACCCAGGAGGGAGCTTTGA<br>Reverse: ATGGCCTGCACGAAGAATCC	131
CAB1	Forward: GAATCTCGCCGACCACCTC<br>Reverse: GGCAAAACTAGCGAGGGAAAGT	100
CAB1B	Forward: GGGTCTCCATGAGGAAGGTC<br>Reverse: TGGGAATTCGCCAGTCAAGT	134
CAB3	Forward: AAGCCCAACTCCAGTTTCCTC<br>Reverse: TACTTGACACGCTCTGGGC	122
CAB3C	Forward: GGGTGCCCTTCAGGATGTAA<br>Reverse: CAGTGTCCCAACCGTAGTCA	188
CAB96	Forward: CTATTTGGGAAACCCGAGCC<br>Reverse: GGTCCTCCGGCAATTCTACAG	108
Carbohydrate metabolism		
BGLU11	Forward: GGCCTGATAATGCACCCCAT<br>Reverse: GCTTTGCTTCCTGCTCTGTG	100
CA	Forward: ACCCCGAATGCGTGTTTATG<br>Reverse: ATATTCAACAGCGGACCCGA	106
CA1	Forward: GCGAAACAAACGGAGCCTAA<br>Reverse: CAACAGCAGATCCAACCCCA	126
LDHA	Forward: TTATGGTGGTGTCCAACCCG<br>Reverse: AGAACCGGAACCTGGAGCTA	120
GAD	Forward: GAATACCATCTGCGTTGCGG<br>Reverse: GAGTGTCCCAGCCTGTGATT	116
GAPC	Forward: CTAGGGCTGTTGGGATGGTG<br>Reverse: CCGGTTGAGGTCAGGAAACA	104
PGAM	Forward: TGTGGAAGGCGAGAAGAACC<br>Reverse: CCAGCTCGGTCGTCTTGTTA	140
fatty acids metabolism		
BEAT	Forward: CCGCTAACCGGTCCATTAGG<br>Reverse: CGTTGCACGAACATCCATCC	109
BPS	Forward: ATCGGGGCAAACCCTAACAC<br>Reverse: ATCTCGTAGAAGTGGGCCGT	116
PED1	Forward: TGCCCCTCAGAACAGTCAAC<br>Reverse: CGCCAATGATGTCCAACGAC	138
PLDZETA1	Forward: TCGGAAGTCAGCAAGCCATT<br>Reverse: TGCTTCACCATCACGTAGCC	184
Cytochrome P450		
CYP75B2	Forward: TTCGTTCCGTTGCTAGAGCC<br>Reverse: TCGCCTTGTGATCCTCCAAC	112
CYP81E1	Forward: TGATCAAGCTGGCCGAGAAG<br>Reverse: AAACTCGGGGTCTGTCTCCT	149
**Plant growth (6)**		
CYP96A15	Forward: AAGAGCTTGGGACACCTTCG<br>Reverse: ATTGTTTCGGTCAGCCCTGT	148
EMS1	Forward: TCCTCAAGACCCTCTCCTCG<br>Reverse: GCTATCCTCATTCGCCTGCT	184
GEM	Forward: ATCACGCAAGGAACGAAGGT<br>Reverse: CGTTCCAGCTATGGGTCCAG	141
MIZ1	Forward: GGAATGGGGTTGGTTCGGAT<br>Reverse: AACCCCGTACCCTGACTTCT	120
SBTI1.1	Forward: CATGTACGCTGCAATGTCGG<br>Reverse: CGTCTTCGATAGGGGAGTGC	164
ZOG1	Forward: CTAACGCCGAGTCCTACACC<br>Reverse: CCTTCAAGCGAAGGGATGGT	121
**Stress response (12)**		
Heat shock protein and cold responsive protein		
HSP70	Forward: GACGACGCCTTCTTACGTGG<br>Reverse: ATCGAAGACGGTGTTGACGG	100
HSC-I	Forward: GCTACCTCCAGCCGACAAAA<br>Reverse: TCCATCGAACCACCATCAGC	186
HSC-2	Forward: CCAATGGCTTGATGGCAACC<br>Reverse: AACTTCATCGTCCATCGGGG	145
COR413IM1	Forward: TGACCACCAGCGAGAAGAAG<br>Reverse: TTGCAGTCCCCTTGGTTAGC	168

CRPK1	Forward: TGTAGCTAGAGGGCTCGCAT<br>Reverse: CTGGAATCAACTTGGCCAGC	140
BAP1	Forward: GAAGTCCACGAGAGCGGATT<br>Reverse: CGGGTGTGTAGTCTCCCAAG	188
Transcription factor		
ERF5	Forward: GGTGACGGAGAGGAGACACT<br>Reverse: ATCAACCGCCGTGTCATAGG	127
ERF020	Forward: ACGACTCTGGCTCGGTACTT<br>Reverse: AAGGCAGCATCAGAGGGAAG	122
MYB4	Forward: CGTGAAACTTGGCGGCTACT<br>Reverse: GATGGGTGTGCCAGTGATTC	143
MYB14	Forward: TCCTCAGCCACATGACACAG<br>Reverse: GAGTCCGCGTTTTCGCTTAC	192
NAC071	Forward: AACGGGACATGGAGTGGTTC<br>Reverse: GTAGCCTTCCAGTACCCAGC	100
WRKY24	Forward: CAATGAAGCGCCACGAAGAG<br>Reverse: GTCCTCTGACCTCCTCGACT	179

Table S2: Summary of sequencing data of *H. perforatum* transcriptome

	15°C	22°C
**Unfiltered data**		
Data of reads number (million)	39.44	45.60
Reads length	150	150
GC (%)	51.40	51.30
Data of reads number×read length (million)	5,916	6,840
Q20(%)	96.97	97.68
Q30(%)	92.26	93.50
**Filtered data[1]**		
Data of reads number (million)	39.36	45.54
Data of reads number×read length (million)	5,879	6,802
GC (%)	51.38	51.29
Q20(%)	97.06	97.75
Q30(%)	92.38	93.59
**Mapped data[2]**		
Data of unique mapped reads (million)	28.60	33.37
Data of multiple mapped reads (million)	2.48	2.90
Mapping ratio (%)	78.96	79.64
**Compiled data**		
Total number of unigenes	44,776	
Total Length (bp) (million)	44.86	
N50 (bp)	1,694	
Max length (bp)	15,636	
Min length (bp)	201	
Average Length (bp)	1,001	
GC content (%)	46.00	

[1] Reads with a quality score < 30 and length < 60 bp were excluded;

[2] Mapping ratio = (Unique mapped reads + Multiple mapped reads) / Filtered reads

Table S3: Database searches for collected *H. perforatum* nucleotide sequences

BLASTx searching against specific platforms	Values	Percentage (%)
NR	29,475	65.83
SwissProt	19,384	43.29
KOG	16,057	35.86
KEGG	27,680	61.82
GO	12, 038	26.88

Table S4: Photosynthesis genes differentially expressed at 15 versus 22℃

Gene name	Protein name	$\log^2$ Ratio (15℃RPKM/22℃RPKM)
CAB1	Chlorophyll a-b binding of LHCII type 1 protein	1.18
CAB1B	Chlorophyll a-b binding protein 1B, chloroplastic	1.26
CAB3	Chlorophyll a-b binding protein 3, chloroplastic	1.13
CAB3C	Chlorophyll a-b binding protein 3C, chloroplastic	1.46
CAB96	Chlorophyll a-b binding protein AB96	1.16
CAB	Chlorophyll a-b binding protein, chloroplastic	1.42
ELI_PEA	Early light-induced protein, chloroplastic	1.97
ELIP2	Early light-induced protein 2, chloroplastic	-1.80
OHP2	Light-harvesting complex-like protein OHP2, chloroplastic	1.60
RBCS-C	Ribulose bisphosphate carboxylase small chain C, chloroplastic	1.45
RCA	Ribulose bisphosphate carboxylase/oxygenase activase, chloroplastic	-1.23
RBCS-8B	Ribulose bisphosphate carboxylase small chain 8B, chloroplastic	-2.69

Table S5: Carbohydrate metabolism genes differentially expressed at 15 versus 22℃

Gene name	Protein name	$\log^2$ Ratio (15℃RPKM/22℃RPKM)
CA	Carbonic anhydrase, chloroplastic	1.43
CA1	Carbonic anhydrase 1	1.23
LDHA	L-lactate dehydrogenase A	2.47
PGAM	Phosphoglycerate mutase-like protein	1.31
AGLU2	Alpha-glucosidase 2	1.16
BGLU11	Beta-glucosidase 11	1.38
GINT1	Glucosamine inositolphosphorylceramide transferase 1	-1.18
GAD	Glutamate decarboxylase	1.15
GAPC	Glyceraldehyde-3-phosphate dehydrogenase, cytosolic	1.84
NonBGLU	Non-cyanogenic beta-glucosidase	1.08
TOGT1	Scopoletin glucosyltransferase	1.14
GAE6	UDP-glucuronate 4-epimerase 6	-1.33
USP1	UDP-sugar pyrophosphorylase 1	-1.30

Table S6: Fatty acids metabolism genes differentially expressed at 15 versus 22℃

Gene name	Protein name	log_2 Ratio (15℃$_{RPKM}$/22℃$_{RPKM}$)
FAD3	Acyl-lipid omega-3 desaturase (cytochrome b5), endoplasmic reticulum	1.04
YLR118C	Acyl-protein thioesterase 1	1.59
abhd17c	Alpha/beta hydrolase domain-containing protein 17C	-1.29
HOS3	Elongation of fatty acids protein 3-like	1.81
FAR3	Fatty acyl-CoA reductase 3	-1.11
MGD3	Monogalactosyldiacylglycerol synthase 3, chloroplastic	1.22
At1g06800	Phospholipase A1-Igamma1, chloroplastic	2.41
At4g16820	Phospholipase A1-Ibeta2, chloroplastic	1.87
PLDZETA1	Phospholipase D zeta 1	1.69
PLD1	Phospholipase D alpha 1	1.27
SQD2	Sulfoquinovosyl transferase SQD2	1.11
LIP2	Triacylglycerol lipase 2	-1.11
BEAT	Acetyl-CoA-benzylalcohol acetyltransferase	1.45
BPS	2,4,6-trihydroxybenzophenone synthase	1.74
PED1	3-ketoacyl-CoA thiolase 2, peroxisomal	1.04

Table S7: Cytochrome P450 genes differentially expressed at 15 versus 22℃

Gene name	Protein name	log_2 Ratio (15℃$_{RPKM}$/22℃$_{RPKM}$)
CYP71A6	Cytochrome P450 71A6	0.08
CYP71A9	Cytochrome P450 71A9	1.59
CYP78A5	Cytochrome P450 78A5	-2.31
CYP82A1	Cytochrome P450 82A1	2.17
CYP82A3	Cytochrome P450 82A3	-1.41
CYP89A9	Cytochrome P450 89A9	1.94
CYP86B1	Cytochrome P450 86B1	2.82
CYP90B1	Cytochrome P450 90B1	-1.11
CYP94B1	Cytochrome P450 94B1	1.79
CYP96A15	Alkane hydroxylase MAH1	1.37
CYP714C2	Cytochrome P450 714C2	1.22
CYP81D1	Cytochrome P450 81D1	1.86
CYP81E8	Cytochrome P450 81E8	0.53
CYP716B1	Cytochrome P450 716B1	1.22
CYP736A12	Cytochrome P450 CYP736A12	1.04
CYP749A22	Cytochrome P450 CYP749A22	-1.33
CYP82D47	Cytochrome P450 CYP82D47	1.80
CYP75B2	Flavonoid 3'-monooxygenase	1.19
CYP93B1	Licodione synthase	1.50
CYP81E9	Isoflavone 3'-hydroxylase	4.31

CYP81E1	Isoflavone 2'-hydroxylase	1.07

Table S8: Morpho-physiological traits genes differentially expressed at 15 versus 22℃

Gene name	Protein name	$\log^2$ Ratio (15℃$_{RPKM}$/22℃$_{RPKM}$)
ZOG1	Zeatin O-glucosyltransferase	-1.29
DCR	BAHD acyltransferase DCR	-1.33
CYP96A15	Alkane hydroxylase MAH1	1.37
MIZ1	Protein MIZU-KUSSEI 1	-0.09
GEM	GLABRA2 expression modulator	-1.02
CET1	CEN-like protein 1	1.54
FPF1	Flowering-promoting factor 1	-3.26
FTIP1	FT-interacting protein 1	1.47
HD3B	Protein HEADING DATE 3B	-1.25
SWC6	SWR1 complex subunit 6	-1.23
SBT1.9	Subtilisin-like protease SBT1.9	1.07
SBT3.18	Subtilisin-like protease SBT3.18	2.69
SBTI1.1	Subtilisin-like protease SBT1.1	2.17
EMS1	Leucine-rich repeat receptor protein kinase EMS1	2.19
MSP1	Leucine-rich repeat receptor protein kinase MSP1	1.04

Table S9: Heat shock protein and cold responsive protein genes differentially expressed at 15 versus 22℃

Gene name	Protein name	$\log^2$ Ratio (15℃$_{RPKM}$/22℃$_{RPKM}$)
HSP15.7	15.7 kDa heat shock protein, peroxisomal	-2.39
HSP17.3-B	17.3 kDa class I heat shock protein	-1.60
HSP17.4B	17.4 kDa class III heat shock protein	-2.99
HSP17.9-D	17.9 kDa class II heat shock protein	-2.60
HSP18.2	18.2 kDa class I heat shock protein	-1.61
HSP22.0	22.0 kDa class IV heat shock protein	-1.83
HSP70-8	Heat shock 70 kDa protein 8	-1.15
HSP70	Heat shock cognate 70 kDa protein	-1.64
HSC-I	Heat shock cognate 70 kDa protein 1	-3.68
HSC-2	Heat shock cognate 70 kDa protein 2	-3.33
HSF30	Heat shock factor protein HSF30	-1.76
HSP81-1	Heat shock protein 81-1	-1.02
HSP83A	Heat shock protein 83	-2.73
HSP21	Small heat shock protein, chloroplastic	-2.69
HSA32	Protein HEAT-STRESS-ASSOCIATED 32	-1.57
BAP1	BON1-associated protein 1	1.43

COR413IM1	Cold-regulated 413 inner membrane protein 1, chloroplastic	1.29
CRPK1	Cold-responsive protein kinase 1	1.22

Table S10: Transcription factors differentially expressed at 15 versus 22℃

Gene name	Protein name	log^{2} Ratio (15℃$_{RPKM}$/22℃$_{RPKM}$)
**Myb family transcription factor**		
PHL5	Myb family transcription factor PHL5	-1.27
MYB2	Transcription factor MYB2	-3.01
MYB3	Transcription factor MYB3	-1.05
MYB4	Transcription factor MYB4	2.48
MYB14	Transcription factor MYB14	1.40
MYB17	Transcription factor MYB17	1.82
MYB48	Transcription factor MYB48	10.44
MYB52	Transcription factor MYB52	-1.02
MYB62	Transcription factor MYB62	-1.36
MYB73	Transcription factor MYB73	-1.08
MYB78	Transcription factor MYB78	1.53
MYB108	Transcription factor MYB108	1.15
**MADS-box transcription factor**		
AGL80	Agamous-like MADS-box protein AGL80	-1.32
SVP	MADS-box protein SVP	-1.26
CMB1	MADS-box protein CMB1	-2.85
MADS50	MADS-box transcription factor 50	-2.09
**Transcription factor bHLHs**		
BHLH62	Transcription factor bHLH62	-1.10
BHLH92	Transcription factor bHLH92	1.11
BHLH96	Transcription factor bHLH96	-1.30
BHLH154	Transcription factor bHLH154	-1.35
BHLH162	Transcription factor bHLH162	2.64
**Transcription factor TCPs**		
TCP1	Transcription factor TCP1	-1.60
TCP12	Transcription factor TCP12	-2.00
TCP20	Transcription factor TCP20	-1.00
**WRKY transcription factor**		
WRKY7	Probable WRKY transcription factor 7	1.56
WRKY40	Probable WRKY transcription factor 40	2.16
WRKY41	Probable WRKY transcription factor 41	2.18
WRKY53	Probable WRKY transcription factor 53	3.02
WRKY72	Probable WRKY transcription factor 72	1.53
WRKY6	WRKY transcription factor 6	1.11
WRKY28	WRKY transcription factor 28	1.75
WRKY55	WRKY transcription factor 55	2.83

WRKY24	WRKY transcription factor WRKY24	1.52
**bZIP transcription factor**		
BZIP44	bZIP transcription factor 44	-1.12
BZIP61	Basic leucine zipper 61	-1.04
TGA10	bZIP transcription factor TGA10	-1.37
**Ethylene-responsive transcription factor**		
At1g50680	AP2/ERF and B3 domain-containing transcription factor At1g50680	-0.52
At1g51120	AP2/ERF and B3 domain-containing transcription factor At1g51120	-1.32
ERF2	Ethylene-responsive transcription factor 2	4.72
ERF2	Ethylene-responsive transcription factor 2	1.93
ERF5	Ethylene-responsive transcription factor 5	1.89
ABR1	Ethylene-responsive transcription factor ABR1	1.32
ERF003	Ethylene-responsive transcription factor ERF003	-1.34
ERF013	Ethylene-responsive transcription factor ERF013	-1.22
ERF017	Ethylene-responsive transcription factor ERF017	3.03
ERF020	Ethylene-responsive transcription factor ERF020	4.50
ERF071	Ethylene-responsive transcription factor ERF071	3.06
ERF095	Ethylene-responsive transcription factor ERF095	4.00
ERF109	Ethylene-responsive transcription factor ERF109	2.48
**NAC domain-containing protein**		
NAC014	NAC domain-containing protein 14	1.10
NAC043	NAC domain-containing protein 43	-1.16
NAC048	NAC domain-containing protein 48	2.19
NAC071	NAC domain-containing protein 71	2.54
NAC087	NAC domain-containing protein 87	1.15
NAC090	NAC domain-containing protein 90	4.53
NAC100	NAC domain-containing protein 100	-0.76
